# Use of Salt-Restriction Spoons and Its Associations with Urinary Sodium and Potassium in the Zhejiang Province of China: Results of a Population-Based Survey

**DOI:** 10.3390/nu13041047

**Published:** 2021-03-24

**Authors:** Xiaofu Du, Di Zhao, Megan E. Henry, Le Fang, Jianwei Xu, Xiangyu Chen, Jie Zhang, Yamin Bai, Jing Wu, Jixiang Ma, Jieming Zhong, Min Yu, Lawrence J. Appel

**Affiliations:** 1 Department of Chronic Disease Prevention and Control, Zhejiang Provincial Center for Disease Control and Prevention, No. 3399 Binsheng Road, Hangzhou 310051, China; xfdu@cdc.zj.cn (X.D.); lef@cdc.zj.cn (L.F.); xychen@cdc.zj.cn (X.C.); jiezhang@cdc.zj.cn (J.Z.); jmzhong@cdc.zj.cn (J.Z.); 2 Welch Center for Prevention, Epidemiology, and Clinical Research, Johns Hopkins University, No. 2024 E. Monument Street, Baltimore, MD 21287, USA; dizhao@jhu.edu (D.Z.); mhenry16@jhu.edu (M.E.H.); 3 National Center for Chronic and Noncommunicable Disease Control and Prevention, Chinese Center for Disease Control and Prevention, No. 27 Nanwei Road, Beijing 100050, China; xujianwei@ncncd.chinacdc.cn (J.X.); baiyamin@ncncd.chinacdc.cn (Y.B.); wujing@chinacdc.cn (J.W.); 4 Shandong Center for Disease Control and Prevention, No. 16992 Jingshi Road, Jinan 250014, China; majix@163.com

**Keywords:** hypertension, urinary sodium and potassium excretion, salt-restriction spoon, knowledge, attitude and behavior

## Abstract

In China, a major source of sodium is salt added during cooking. In this context, use of a salt-restriction spoon (SRS) has been promoted in public health campaigns and by health care providers. To describe use of and factors associated with SRS use, knowledge of correct use, and actual correct use. This study is a population-based, representative survey of 7512 residents, aged 18 to 69 years, of China’s Zhejiang Province. The survey, which was conducted in 2017 using a multistage random sampling strategy, collected demographic information, SRS use, and physical measurements; a 24-h urine collection was obtained from 1,496 of the participants. The mean age of the participants was 44.8 years, 50.1% were females, and over 1/3 (35.3%) were classified as hypertensive. Mean 24-h urinary sodium and potassium excretions were 167.3(72.2) mmol/24 h and 38.2(18.2) mmol/24 h, respectively. Only 12.0% (899/7512) of participants once used or were currently using SRS; of the 899 users, 73.4% knew how to use the SRS correctly, and just 46.5% actually used it correctly. SRS use was more commonly associated with behavioral factors rather than socio-demographic factors. Initiation of SRS use by health care providers was associated with correct technical knowledge of SRS. Lower sodium-to-potassium ratio was associated with SRS use, while SRS use was not associated with urinary sodium and potassium excretion. Use of SRS was uncommon in Zhejiang Province of China. Given that a common source of sodium in China is salt added during cooking, use of SRS is an appealing strategy, ideally as part of a multi-component campaign.

## 1. Introduction

A high-salt diet, which is causally related to the development of hypertension, is among the leading four risk factors contributing to deaths and DALYs [1]. In China, the last (2012–2015) national hypertension survey showed the overall prevalence of hypertension and prehypertension was 23.2% and 41.3%, respectively, which mean that approximately 244.5 million and 435.3 million individuals in the Chinese adult population have hypertension or prehypertension [2]. In addition, consumption of salt is among the highest in the world. Average daily intake of salt (sodium chloride) in China is 10.5 g/d [3], which greatly exceeds the recommended limit of 6 g/d set by the Chinese Dietary Guidelines [4] and the recommended limit of 5 g/d set by the World Health Organization (WHO) [5]. Furthermore, average daily potassium intake in China, estimated from urinary potassium excretion, is less than half of the WHO recommendation of >3.5 g/day [6]. Strategies to reduce salt intake and increase potassium intake are critical to preventing and controlling hypertension and its cardiovascular disease (CVD) consequences [7,8,9].

Use of Salt-Restriction Spoons (SRS) has been advocated as a means to lower salt intake in China and other Asian countries where a predominant source of sodium is the addition of salt during cooking. Around 75.8% of the sodium in the diet of Chinese residents comes from household cooking salt, followed by high-salt seasoning [10]. In contrast, the predominant source in western countries is the addition of salt during food manufacturing. The Chinese Guidelines for Prevention and Treatment of Hypertension emphasized the need to implement salt reduction interventions in patients with hypertension or with kidney disease, children, pregnant women and the elderly, and it was recommended to use a quantitative salt spoon as much as possible during cooking to serve as a warning [10]. SRS differ in design and capacity (e.g., 2, 3, and 6 gm), with each designed to assist users in reducing salt used during cooking (Figure S1). Many local governments in China, including the Zhejiang Province, have distributed millions of spoons; in addition, physicians have also encouraged SRS use.

In this context, we conducted a cross-sectional survey in Zhejiang Province in 2017. The objective of this study is to describe use of SRS, knowledge of correct use, and actual correct use. Such information can guide and refine public health campaigns designed to lower salt intake in China.

## 2. Materials and Methods

Data were collected from a cross-sectional survey conducted in Zhejiang Province of China in 2017. Zhejiang Province is located in southeastern China and is an economically developed province with a population of approximately 55 million. In 2019, the GDP (Gross Domestic Product) of Zhejiang Province ranked fourth in the country, and the per capita GDP was 16,000 US dollars, the second highest in China. Therefore, public health expenditures and per capita medical and health resources are among the highest in China. Permission to undertake the survey was obtained from China CDC. The Zhejiang Provincial CDC Ethics Review Committee approved the project. All participants provided written, informed consent.

### 2.1. Participant Recruitment

Participants were aged 18 to 69 years without disability and psychiatric disorders and living in the selected areas for at least six months before the investigation. A stratified multistage random sampling method was used. Briefly, to enhance generalizability, the study included two rural areas and three urban areas. Each of the 75 villages or neighborhood committees randomly selected and subsequently recruited qualified residents in the area. In randomly selected communities, a roster was established through the electronic health records of local residents which was divided into 10 levels according to age and gender, and survey subjects were randomly selected in each level. The provincial sample consisted of 7512 residents, of which a sex- and age-stratified random sample of 1496 responders collected a 24-h urine sample for urinary sodium and potassium excretion. Methods for sample size calculation and sample selection are described in detail elsewhere [11].

### 2.2. Data Collection

Trained data collectors asked participants to answer questions on socio-demographic characteristics; history of hypertension, diabetes and CVD; lifestyle habits of smoking, alcohol use, and physical activity. Former smoker was defined as >100 cigarettes smoked but no current consumption, and current smoker was defined as an average of >1 cigarette per day for 6 consecutive months; participants were otherwise defined as nonsmokers. Self-reported physical activity was ≥150 min per week of moderate-intensity or a combination of moderate- and high-intensity exercise or ≥75 min per week of high-intensity exercise. Alcohol consumption was defined as a response of ≥1 time a week in the past year; alcoholic beverages included beer, liquor, red wine, and rice wine. Brachial systolic blood pressure (SBP) and diastolic blood pressure (DBP) were measured according to internationally accepted standards; we obtained triplicate measurements with the participant seated after 5 min of rest [12]. Measurements were recorded at least one minute apart using a validated automatic electronic sphygmomanometer (HEM-7071, Omron Corp., Japan) with an appropriately sized cuff; the average value of the three measurements was used in analysis. Hypertension was defined as mean SBP ≥140 mm Hg and/or mean DBP ≥90 mm Hg and/or self-reported use of antihypertensive medication within two weeks [13]. The data collectors also measured weight and height, and specific measurement methods were described in detail elsewhere [11].

One 24-h urine collection was used to estimate urinary sodium and potassium excretion in a subset of 1572 participants. Each of these participants received information on how to collect a high quality sample; they were also provided the requisite equipment, including a 3 L standard urine collection container [14]. Data collectors recorded the start and end times of the 24-h urine collection and measured its volume. Indicators of an incomplete collection were length of collection time <22 h or >28 h, urine volume < 500 mL, or creatinine excretion ±2 standard deviations outside of the sex-specific mean [15,16]. An aliquot of the sample was shipped to a central lab (KingMed Diagnostics Laboratory Inc., Hangzhou, China) for measurement of sodium, potassium, and creatinine. An ion selective electrode method was used for sodium and potassium analyses (C16000, Abbott Corp., America) and a picric acid method for creatinine (C501, Roche Cob as Corp., Switzerland). Urinary excretion was the cross-product of the concentration of the analyte multiplied by the 24-h urine volume. Of participants selected for the 24-h urine collection, 1496 (95% of 1572) returned a complete specimen.2.3 SRS status

Participants were asked about their knowledge, attitude, and behavior (KAB) related to salt (File S1). We used the World Health Organization/Pan American Health Organization (WHO/PAHO) protocol for population level sodium assessment, which we adapted based on a review of the literature and consultations with public health practitioners and experts [17,18,19]. A total of 19 questions were selected.

We asked three related and hierarchical questions about SRS. To enhance data quality, we standardized data collection methods and trained the data collectors.

The purpose of the first question, “SRS Adoption,” was to understand whether the respondent once used or was using SRS during cooking, no matter how they used it. The question was “Did you use or are you using an SRS?” The data collector had the option of confirming an unclear response by showing the respondent an SRS or viewing the spoon used by the respondent. If the response to the first question was “yes,” the respondent continued to the second question.

The purpose of the second question, “SRS Knowledge,” was to determine if the respondent knew how to use the SRS technically, independent of whether they actually used it correctly. The question was, “Do you know how to use SRS correctly?” The respondent could describe or demonstrate how to use it. The data collector used the response to the question and direct observations to provide an answer. Features of correct use included using the scale on the SRS, distinguishing SRS from an ordinary spoon, and adding salt so that the level was flat and no higher than the upper edge of the SRS. If the answer to the second question was “yes,” the respondent continued to the third question.

Technical knowledge of correct use (a “yes” response to the second question) does not ensure correct use. Therefore, we asked a third question, “Do you usually use the SRS correctly in daily cooking?” The respondent could describe how he/she used the SRS during routine food preparation. Again, the data collector used the respondent’s answer to the third question and additional queries to provide a response (yes/no).

### 2.3. Statistical Analysis

The characteristics of the participants were summarized as proportions and means (SD, standard deviation). ANOVA for continuous variables and categorical variables was used to compare the general characteristics, KAB variables, SRS characteristics, and 24-h urinary electrolyte excretion, by response to the three SRS questions. Multivariable linear regression was used to assess the associations of SRS responses with 24-h urinary sodium/creatinine molar ratio (mmol/mmol), potassium/creatinine molar ratio (mmol/mmol), and sodium-to-potassium ratio (mmol/mmol), and age, gender, and region that may affect urine electrolyte content largely were preliminarily adjusted. Statistical analyses were performed with SPSS for Windows (Version 26, SPSS Inc., Chicago, IL, USA). *p* values < 0.05 were considered statistically significant.

## 3. Results

### 3.1. Study Participants

The average age of the participants was 44.8(14.0) years old, 50.1% were females, and 98.1% were Han ethnicity (Table 1). Mean SBP and DBP were 129.5(19.1) mm Hg and 80.4(10.9) mm Hg, respectively, and over 1/3 (35.3%) of participants were classified as hypertensive.

Of 7512 participants, only 12.0% (899) once used or were currently using SRS. Of these 899 participants, 73.4% (660) knew how to use the SRS correctly; 418 persons actually used the SRS correctly (46.4% of all users, and 63.3% of those who knew how to use it).

Participants who once used or were currently using SRS generally had a higher education level than those who had not used SRS. Participants from urban areas were more likely to have used SRS and have a higher rate of correct technical knowledge than participants in rural areas. No sociodemographic variables were significantly associated with correct vs non-correct use of SRS (Table 1).

### 3.2. Additional Characteristics of SRS Users

Of the 899 participants who use or used SRS, 59.7% of participants purchased the SRS without receiving advice from their healthcare provider, 22.7% of participants purchased the SRS after receiving advice from their health care provider, and 16.6% of participants received it as a free giveaway. Of 204 individuals who received advice from their health care provider, 92.6% knew how to use the SRS correctly; in contrast, just 68% of the 527 who purchased the SRS without healthcare provider advice knew how to use it correctly. Size of the SRS was not significantly associated with knowledge of correct SRS or actual correct use. (Table 2).

### 3.3. Knowledge, Attitude and Behavior (KAB)

For virtually all KAB questions, most participants stated that they had knowledge related to hypertension, salt, and their adverse effects. Notable exceptions were lack of knowledge of the recommended daily salt intake (less than 6 g salt per day) at 38.9%; lack of knowledge on low-sodium salt, at 30.0%; and lack of knowledge that low-sodium salt lowered blood pressure, at 20.8%. Each of the four attitude questions related to use of low-sodium salt or nutrition-labeling, with participants indicating approval ranging from 72.6% to 88.2%. However, few participants indicated that they paid attention to nutrition-labeling (22.1%), and even fewer participants actually used a low-sodium salt product (15.7%). Uniformly, SRS users reported significantly higher levels for each KAB question than those who did not use the SRS; likewise, there was a similar pattern for responses, stratified by SRS knowledge status. In contrast, there were fewer significant differences in KAB, stratified by correct SRS use. Overall, while there were many statistically significant differences, the absolute differences were often modest in size (Table 3).

### 3.4. Associations of 24-h Urinary Sodium, Potassium Excretion, and Sodium-to-potassium Ratio with SRS Status

Mean(SD) 24-h urinary sodium excretion of the 1496 participants who provided 24-h urine collections was 167.3(72.2) mmol/24 h, corresponding to 9.8(4.2) grams/d; 96.0% of participants exceeded the WHO recommended maximum level of 5 g/d, and 63.3% exceeded the Chinese Nutrition Society recommended maximum level of 6 g/d. Mean (SD) 24-h urinary potassium excretion was 38.2(18.2) mmol/24 h, corresponding to 1.5 g(0.7) grams/d. Participants who used or were currently using SRS had significantly lower sodium-to-potassium ratio compared to non-users. The urinary sodium-to-potassium ratio was lower among those who knew how to use the SRS correctly. There were no significant differences by actual correct use of SRS (Table 4).

## 4. Discussion

Salt reduction is a global priority, with the ultimate goal of reducing the public health burden of non-communicable diseases. As early as the 1970s, some economically developed countries had implemented salt-reduction strategies. In 2006, the WHO Expert Committee on salt reduction recommended that every country should establish its own strategy. Now, more than 80 countries have national salt-reduction strategies in place. The majority of strategies are multifaceted, including sodium-reduction plans and actions in the food industry, consumer education, warning signs and front-of-pack labelling on foods, the promotion of low sodium diets, potassium-enriched salt, and comprehensive interventions in public institutions. While the main dietary source of salt in Western countries is processed foods, home cooking is the main source of dietary salt in China. Hence, strategies to reduce salt during cooking are critical to achieving population-wide reduction in salt intake. With this goal in mind, use of SRS in China has been commonly implemented as part of a multi-component approach to lower salt intake during cooking; other components include health education and use of potassium-enriched, low-sodium salt.

Between 2007 and 2009, Beijing, Shanghai, and Guangzhou distributed free SRS, with a capacity of 2 g and 6 g, to thousands of households. From 2011 through 2016, more than 13 million SRS were widely distributed to households in Shandong Province [20]. Through mail, community distribution, special events, and other methods, the government strongly encouraged its citizens to develop a healthy lifestyle of less than “6 g of salt per person per day.” In addition, SRS was also distributed with small packages of table salt sold in markets. Subsequently, the “China Healthy Lifestyles for All” campaign managed by the China CDC was implemented by the 4-level CDC (the national, provincial, municipal, and county level), and became the main platform for the promotion of SRS [21].

Several studies have suggested that SRS might reduce sodium intake and blood pressure. A meta-analysis suggests that the adoption of SRS could result in a mean decrease of 1.46 g and a maximum decrease of 2.36 g in daily salt intake among Chinese adults [22]. Comprehensive interventions not limited to SRS were associated with significant decreases in dietary salt intake and in a reduction in blood pressure [20,23,24]. Few reports on the adoption, correct technical knowledge, and correct use of SRS at the population level have previously been published. To our knowledge, this study is the first large-scale investigation of the characteristics and risk factors of SRS use.

In this population-based survey in the Zhejiang Province of China conducted in 2017, only 12.0% of participants once used or were currently using SRS; of these, 73.4% correctly knew how to use the SRS, but only 46.5% actually used it correctly. Other notable findings were that SRS was more commonly associated with behavioral factors than with socio-demographic factors, and that knowledge of correct SRS use was associated with initiation by health care provider. The distribution of SRS does not mean that it will be used and adopted by the consumers and subsequently bring the corresponding benefits. Our research was consistent with the results of similar studies and found that the correct use rate was the lower than the adoption or awareness rate [25]. The survey showed that most SRS was obtained from product giveaway or self-consumption, without technical knowledge of correct use provided from healthcare providers. Therefore, consumers may lack the motivation to use SRS, knowledge of correct use, and the positive feedback of the health benefits from using SRS. The Health Belief Model postulates that when an individual perceives a threat or hazards from a disease or unhealthy lifestyle, and perceives benefits from preventive actions exceed the barriers, then the individual is likely to take preventive action [26,27]. A study on SRS suggested that the perceived severity of high-salt diet and perceived benefits of SRS, as well as perceived barriers influence the adoption of SRS [28]. Therefore, consumers should understand the hazards of excessive salt intake and receive technical knowledge of SRS during distribution in order to better stimulate and maintain the use of SRS [29].

Other research has shown that the reduction in salt intake was evident for people using SRS compared with nonusers for cooking (−3.49 g versus −2.22 g) after adjustment of confounding factors [30]. Based on the cross-sectional analysis in our study, we did not observe linear, dose-response association of 24-h urinary sodium/creatinine molar ratio or potassium/creatinine molar ratio with SRS status among participants, although a positive association between sodium-to-potassium ratio and SRS status was observed. The previous study indicated that a reduction in the 24-h urinary sodium-to-potassium ratio attributable to salt restriction resulted in a blunted deterioration of BP. Therefore, health benefits associated with SRS use may be through the reduction of the ratio of sodium-to-potassium.

Our study has limitations. First, this study is cross-sectional; hence, while we can document associations but we cannot draw causal inferences. Second, because of cost and the high participant burden, we obtained just one 24-h urine collection from participants [31]; without multiple collections, statistical power is reduced given the large intra-individual variability in urinary sodium excretion. For this reason, it is likely that the strength of the associations of sodium and potassium excretion with SRS use is under-estimated [32]. Third, knowledge, attitudes, and behavior (KAB) were self-reported and tended to be associated with SRS use, i.e., potentially biased by social expectation. Fourth, the study did not distinguish current from prior SRS users.

Our study also has several strengths, including its large and diverse sample size, population-based sampling, extensive quality control with data collection by trained staff, and concurrent collection of blood pressure and 24-h urine specimens (in a subsample). The assessment of sodium and potassium excretion based on 24-h urine collections is considered the gold standard for estimating sodium intake [16].

This study is the first large-scale study on the characteristics and risk factors of SRS use and the association between SRS and 24-h urinary sodium, potassium excretion and blood pressure in China. Findings from this study can be used to improve the effectiveness of SRS, e.g., by encouraging physicians to promote SRS use and reinforce its importance as a means to lower salt intake. Our finding that product giveaways were associated with lower knowledge of correct us highlights the need for concurrent instruction on correct use. As with other behavioral interventions, periodic reinforcement is needed to assure correct and continued lifestyle change.

## 5. Conclusions

This population-based survey in the Zhejiang Province of China conducted in 2017 showed that awareness, adoption and correct use of SRS can be improved in the Chinese population. SRS was more commonly associated with behavioral factors related to salt than with socio-demographic factors, and that technical knowledge of correct SRS use was associated with initiation by a health care provider. Our study is beneficial to objectively review the practical significance and improvement direction of the SRS and is of value for policy makers to develop and implement public health strategies. Use of SRS is an appealing strategy, ideally as part of a multi-component campaign.

## Figures and Tables

**Table 1:** Socio-demographic characteristics of 7512 participants by salt-restriction spoon status.

Characteristic	All ^a^	Using or Used SRS			Know How to Use SRS Correctly			Using SRS Correctly		
	(*n* = 7512)	Yes (*n* = 899)	No (*n* = 6613)	*p* Value	Yes (*n* = 660)	No (*n* = 239)	*p* Value	Yes (*n* = 418)	No (*n* = 242)	*p* Value
Age, year	44.8 (14.0)	43.7 (13.1)	45.0 (14.1)	0.009 *	43.7 (13.0)	43.6 (13.5)	0.97	44.4 (12.9)	42.4 (13.1)	0.06
Gender, *n* (%)				0.21			0.004 *			0.09
Male	3746 (49.9)	466 (51.8)	3278 (49.6)		323 (48.9)	143 (59.8)		215 (51.4)	108 (44.6)	
Female	3766 (50.1)	433 (48.2)	3332 (50.4)		337 (51.1)	96 (40.2)		203 (48.6)	134 (55.4)	
Ethnicity, *n* (%)				0.23			0.94			0.13
Han	7364 (98.1)	877 (97.6)	6487 (98.1)		644 (97.6)	233 (97.5)		405 (96.9)	239 (98.8)	
Others	145 (1.9)	22 (2.4)	123 (1.9)		16 (2.4)	6 (2.5)		13 (3.1)	3 (1.2)	
Education, *n* (%)				<0.001 *			<0.001 *			0.96
<9 years	2281 (30.4)	160 (17.8)	2121 (32.1)		105 (15.9)	55 (23.0)		62 (14.8)	43 (17.8)	
9–12 years	3608 (48.0)	479 (53.3)	3129 (47.3)		342 (51.8)	137 (57.3)		226 (54.1)	116 (47.9)	
>12 years	1620 (21.6)	260 (28.9)	1360 (20.6)		213 (32.3)	47 (19.7)		130 (31.1)	83 (34.3)	
Urbanity, *n* (%)				<0.001 *			<0.001 *			0.10
Urban	3303 (44.0)	479 (53.3)	2821 (42.7)		374 (56.7)	105 (43.9)		247 (59.1)	127 (52.5)	
Rural	4209 (56.0)	420 (46.7)	3789 (57.3)		286 (43.3)	134 (56.1)		171 (40.9)	115 (47.5)	
Hypertension status, *n* (%)				0.90			0.75			0.07
Normotensive	4859 (64.7)	583 (64.8)	4273 (64.6)		426 (64.5)	157 (65.7)		259 (62.0)	167 (69.0)	
Hypertensive	2653 (35.3)	316 (35.2)	2337 (35.4)		234 (35.5)	82 (34.3)		159 (38.0)	75 (31.0)	
Systolic blood pressure, mm Hg	129.5 (19.1)	128.7 (18.5)	129.6 (19.2)	0.19	128.0 (18.3)	130.4 (18.9)	0.09	129.0 (18.2)	126.4 (18.5)	0.08
Diastolic blood pressure, mm Hg	80.4 (10.9)	80.5 (10.7)	80.4 (10.9)	0.92	80.2 (10.6)	81.3 (10.8)	0.16	80.5 (10.6)	79.5 (10.6)	0.25
Body Mass Index, kg/m^2^	23.9 (3.4)	24.1 (3.6)	23.9 (3.4)	0.08	24.0 (3.6)	24.3 (3.7)	0.33	24.2 (3.5)	23.8 (3.6)	0.19
Stroke, *n* (%)	62 (0.8)	6 (0.7)	56 (0.8)	0.58	3 (0.5)	3 (1.3)	0.19	2 (0.5)	1 (0.4)	0.90
Coronary heart disease, *n* (%)	72 (1.0)	9 (1.0)	63 (1.0)	0.89	6 (0.9)	3 (1.3)	0.65	4 (1.0)	2 (0.8)	0.87
Diabetes mellitus, *n* (%)	565 (7.5)	68 (7.6)	497 (7.5)	0.96	57 (8.6)	11 (4.6)	0.043 *	39 (9.3)	18 (7.4)	0.41
Kidney disease, *n* (%)	21 (0.3)	3 (0.3)	18 (0.3)	0.74	3 (0.5)	0 (0.0)	0.30	3 (0.7)	0 (0.0)	0.19
Smoking status, *n* (%)				0.59			<0.001 *			0.46
Never smoked	5372 (71.5)	649 (72.2)	4723 (71.5)		502 (76.1)	147 (61.5)		315 (75.4)	187 (77.3)	
Former smoker	312 (4.2)	40 (4.4)	272 (4.1)		23 (3.5)	17 (7.1)		13 (3.1)	10 (4.1)	
Current smoker	1825 (24.3)	210 (23.4)	1615 (24.4)		135 (20.5)	75 (31.4)		90 (21.5)	45 (18.6)	
Alcohol use status, *n* (%)	2474 (32.9)	302 (33.6)	2172 (32.9)	0.66	203 (30.8)	99 (41.4)	0.003 *	136 (32.5)	67 (27.7)	0.19
Physical activity, *n* (%)	3020 (40.2)	500 (55.6)	2520 (38.1)	<0.001 *	396 (60.0)	104 (43.5)	<0.001 *	260 (62.2)	136 (56.2)	0.13

Samples sizes (*n*), means and prevalence were unweighted. ANOVA was used to compare the characteristics of different SRS status. ^a^ Percentages are column percent. * *p* < 0.05. SRS: Salt-restriction spoon.

**Table 2:** Additional characteristics of the 899 salt-restriction spoon users, *n* (%).

Characteristic	All Who Use or Used SRS ^a^	Know How to use SRS Correctly			Using SRS Correctly		
	(*n* = 899)	Yes (*n* = 660)	No (*n* = 239)	*p* Value	Yes (*n* = 418)	No (*n* = 242)	*p* Value
Self-purchase with healthcare provider advice	204 (22.7)	189 (92.6)	15 (7.6)	<0.001 *	128 (67.7)	61 (32.3)	0.14
Product giveaway	149 (16.6)	102 (68.5)	47 (31.5)	0.13	60 (58.8)	42 (41.2)	0.30
Self-purchase without healthcare provider advice	537 (59.7)	366 (68.2)	171 (31.8)	<0.001 *	228 (62.3)	138 (37.7)	0.54
Other reason	9 (1.0)	3 (33.3)	6 (66.7)	0.006 *	2 (66.7)	1 (33.3)	0.90
2 g Size	267 (29.7)	204 (76.4)	63 (23.6)	0.19	137 (67.2)	67 (32.8)	0.17
3 g Size	208 (23.1)	144 (69.2)	64 (30.8)	0.12	85 (59.0)	59 (41.0)	0.23
6 g Size	396 (44.0)	293 (74.0)	103 (26.0)	0.73	189 (64.5)	104 (35.5)	0.58
Other size	28 (3.1)	19 (67.9)	9 (32.1)	0.50	7 (36.8)	12 (63.2)	0.15

ANOVA was used to compare the characteristics of different SRS status. ^a^ Percentages are row percent. * *p* < 0.05.

**Table 3:** Characteristics of knowledge, attitude, and behavior of 7512 participants by salt-restriction spoon status.

Characteristic	All	Using or Used SRS			Know How to Use SRS Correctly			Using SRS Correctly		
	(*n* = 7512)	Yes (*n* = 899)	No (*n* = 6613)	*p* Value	Yes (*n* = 660)	No (*n* = 239)	*p* Value	Yes (*n* = 418)	No (*n* = 242)	*p* Value
**Knowledge**										
Know the diagnostic criteria of hypertension	3998 (53.2)	602 (67.0)	3396 (51.4)	<0.001 *	471 (71.4)	131 (54.8)	<0.001 *	320 (76.6)	151 (62.4)	<0.001 *
Know the hazards of hypertension	5597 (74.5)	770 (85.7)	4827 (73.0)	<0.001 *	591 (89.5)	179 (74.9)	<0.001 *	380 (90.9)	211 (87.2)	0.13
Know the risk factors of hypertension	5963 (79.4)	808 (89.9)	5155 (78.0)	<0.001 *	614 (93.0)	194 (81.2)	<0.001 *	394 (94.3)	220 (90.9)	0.10
Know less than 6 g salt per day	2923 (38.9)	508 (56.5)	2415 (36.5)	<0.001 *	408 (61.8)	100 (41.8)	<0.001 *	271 (64.8)	137 (56.6)	0.036 *
Know that eating less salt lowers blood pressure	4935 (65.7)	717 (79.8)	4218 (63.8)	<0.001 *	553 (83.8)	164 (68.6)	<0.001 *	361 (86.4)	192 (79.3)	0.018 *
Know the hazards of high salt	5339 (71.1)	765 (85.1)	4574 (69.2)	<0.001 *	591 (89.5)	174 (72.8)	<0.001 *	386 (92.3)	205 (84.7)	0.002 *
Know what kind of people should eat a low-salt diet	5960 (79.3)	813 (90.4)	5147 (77.9)	<0.001 *	617 (93.5)	196 (82.0)	<0.001 *	396 (94.7)	221 (91.3)	0.09
Know low-sodium salt	2256 (30.0)	430 (47.8)	1826 (27.6)	<0.001 *	350 (53.0)	80 (33.5)	<0.001 *	233 (55.7)	117 (48.3)	0.07
Know that low-sodium salt helps control blood pressure	1561 (20.8)	352 (39.2)	1209 (18.3)	<0.001*	300 (45.5)	52 (21.8)	<0.001 *	205 (49.0)	95 (39.3)	0.015 *
**Attitude**										
Approve that low-salt diet should be promoted	6540 (87.1)	846 (94.1)	5694 (86.1)	<0.001 *	642 (97.3)	204 (85.4)	<0.001 *	411 (98.3)	231 (95.5)	0.029 *
Approve low-salt diet	6622 (88.2)	844 (93.9)	5778 (87.4)	<0.001 *	638 (96.7)	206 (86.2)	<0.001 *	405 (96.9)	233 (96.3)	0.68
Approve nutrition labeling of prepackaged food	5641 (75.1)	768 (85.4)	4873 (73.7)	<0.001 *	593 (89.8)	175 (73.2)	<0.001 *	383 (91.6)	210 (86.8)	0.047 *
Believe that nutrition labeling of prepackaged food will help to choose low-salt diet	5456 (72.6)	770 (85.7)	4686 (70.9)	<0.001 *	593 (89.8)	177 (74.1)	<0.001 *	381 (91.1)	212 (87.6)	0.15
**Behavior**										
Self-assessment salt level				<0.001 *			0.029 *			<0.001 *
Not much	2135 (28.4)	289 (32.1)	1846 (27.9)		217 (32.9)	72 (30.1)		153 (36.6)	64 (26.4)	
Moderate	3974 (52.9)	486 (54.1)	3488 (52.8)		366 (55.5)	120 (50.2)		236 (56.5)	130 (53.7)	
Excessive	1400 (18.6)	124 (13.8)	1276 (19.3)		77 (11.7)	47 (19.7)		29 (6.9)	48 (19.8)	
Received publicity or education on low-salt diet	3678 (49.0)	592 (65.9)	3086 (46.7)	<0.001 *	485 (73.5)	107 (44.8)	<0.001 *	321 (76.8)	164 (67.8)	0.011 *
Pay attention to the nutrition label of prepackaged food	1663 (22.1)	381 (42.4)	1282 (19.4)	<0.001 *	335 (50.8)	46 (19.2)	<0.001 *	237 (56.7)	98 (40.5)	<0.001 *
Plan to reduce salt	5861 (78.0)	780 (86.8)	5081 (76.9)	<0.001 *	588 (89.1)	192 (80.3)	<0.001 *	377 (90.2)	211 (87.2)	0.23
Take initiative to reduce salt	4377 (58.3)	709 (78.9)	3668 (55.5)	<0.001 *	565 (85.6)	144 (60.3)	<0.001 *	373 (89.2)	192 (79.3)	<0.001 *
Using or used low-sodium salt	1178 (15.7)	270 (30.0)	908 (13.7)	<0.001 *	232 (35.2)	38 (15.9)	<0.001 *	163 (39.0)	69 (28.5)	0.007 *

ANOVA was used to compare the characteristics of different SRS status. * *p* < 0.05.

**Table 4:** Urinary excretion of electrolytes from 24-h urine specimens by salt-restriction spoon status in subset of participants [*n*=1496].

Characteristic	All	Using or Used SRS			Know How to Use SRS Correctly			Using SRS Correctly		
	(*n* = 1496)	Yes (*n* = 161)	No (*n* = 1335)	*p* Value	Yes (*n* = 116)	No (*n* = 45)	*p* Value	Yes (*n* = 77)	No (*n* = 39)	*p* Value
24-h urinary sodium/creatinine molar ratio (mmol/mmol)	167.3 (72.2)	166.9 (74.7)	167.4 (71.9)	0.37	166.5 (73.5)	167.7 (78.6)	0.87	170.6 (76.6)	158.5 (67.1)	0.39
24-h urinary potassium/creatinine molar ratio (mmol/mmol)	38.2 (18.2)	41.8 (21.8)	37.8 (17.7)	0.88	43.6 (22.5)	37.2 (19.3)	0.17	44.2 (21.8)	42.5 (23.9)	0.52
Sodium-to-potassium ratio (mmol/mmol)	4.9 (2.4)	4.5 (2.1)	5.0 (2.5)	0.029 *	4.3 (1.8)	5.1 (2.5)	0.046 *	4.2 (1.7)	4.3 (2.1)	0.48
24-h urinary creatinine, mmol/24 h	10.1 (4.8)	11.0 (6.0)	10.0 (4.7)	0.010 *	11.0 (6.0)	11.1 (6.2)	0.84	11.3 (6.0)	10.4 (6.0)	0.87
24-h urinary volume, mL/24 h	1449.3 (448.6)	1539.9 (517.8)	1438.4 (438.5)	0.018 *	1564.3 (549.0)	1477.0 (425.8)	0.37	1583.9 (540.9)	1525.6 (569.9)	0.57

Data are presented as mean (SD). ANOVA was used to compare the characteristics of different SRS status after adjusting for age, gender, and urban/rural. * *p* < 0.05.

## Data Availability

The data presented in this study are available on request from the corresponding author. The data are not publicly available due to the project in progress and privacy.

## Supplementary Materials

The following are available online at https://www.mdpi.com/2072-6643/13/4/1047/s1, Figure S1: Common salt-restriction spoon, salt-restriction saltshaker and quantitative saltshakers in China. File S1. Questionnaire about knowledge, attitudes and behaviors related to salt.

## Abbreviations

SRS: salt-restriction spoon; WHO: World Health Organization; KAB: knowledge, attitude and behavior; CVD: cardiovascular diseases; CDC: Center for Disease Control and Prevention; BP: blood pressure; SBP: systolic blood pressure; DBP, diastolic blood pressure; BMI: body mass index; SD: standard deviation; CI, confidence interval; OR, odds ratio.
