# Supplementary material for: Use of Salt-Restriction Spoons and Its Associations with Urinary Sodium and Potassium in the Zhejiang Province of China: Results of a Population-Based Survey
Source: Nutrients. 2021 Mar 24;13(4):1047. doi: 10.3390/nu13041047 (PMC8063796; doi:10.3390/nu13041047)
Supplement: Supplementary file 1 [file nutrients-13-01047-s001.pdf]

Online Supporting Material

Use of Salt-Restriction Spoons and Its Associations with Urinary Sodium and Potassium in the Zhejiang Province of China: Results of a Population-Based Survey

Xiaofu Du [1], Di Zhao [2], Megan E. Henry [2], Le Fang [1], Jianwei Xu [3], Xiangyu Chen [1], Jie Zhang [1], Yamin Bai [3], Jing Wu [3], Jixiang Ma [4], Jieming Zhong [1], Min Yu [1,*] and Lawrence J. Appel [2,*]

[1] Department of Chronic Disease Prevention and Control, Zhejiang Provincial Center for Disease Control and Prevention, No. 3399 Binsheng Road, Hangzhou 310051, China; xfdu@cdc.zj.cn (X.D.); lef@cdc.zj.cn (L.F.); xychen@cdc.zj.cn (X.C.); jiezhang@cdc.zj.cn (J.Z.); jmzhong@cdc.zj.cn (J.Z.)

[2] Welch Center for Prevention, Epidemiology, and Clinical Research, Johns Hopkins University, No. 2024 E. Monument Street, Baltimore, MD 21287, USA; dizhao@jhu.edu (D.Z.); mhenry16@jhu.edu (M.E.H.)

[3] National Center for Chronic and Noncommunicable Disease Control and Prevention, Chinese Center for Disease Control and Prevention, No. 27 Nanwei Road, Beijing 100050, China; xujianwei@ncncd.chinacdc.cn (J.X.); baiyamin@ncncd.chinacdc.cn (Y.B.); wujing@chinacdc.cn (J.W.);

[4] Shandong Center for Disease Control and Prevention, No. 16992 Jingshi Road, Jinan 250014, China; majix@163.com

* Correspondence: myu@cdc.zj.cn (M.Y.); lappel1@jhmi.edu (L.J.A.)

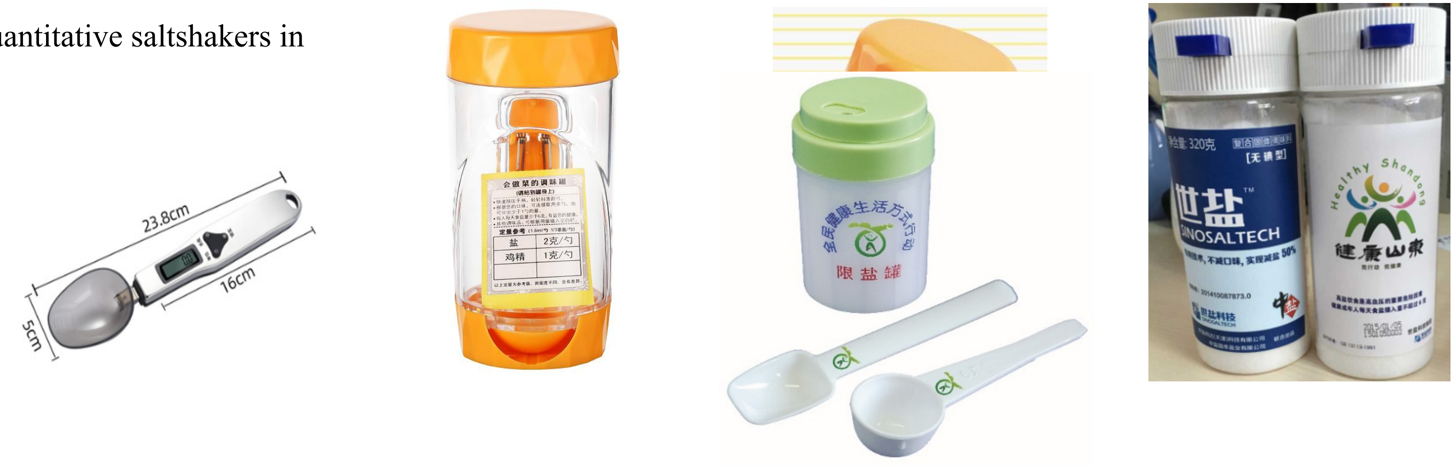

Figure S1. Common salt-restriction spoon, salt-restriction saltshaker and quantitative saltshakers in China.

A **B** **C1** **C2** **D**

A: Distributed salt-restriction spoon and salt-restriction saltshaker by "China Healthy Lifestyles for All" action.

B: Electronic salt spoon, accuracy of 0.1g.

C1-2: Quantitative saltshaker (1g salt) with outlet at the bottom.

D: Quantitative saltshaker (1g salt) with outlet on top.

File S1. Questionnaire about knowledge, attitudes and behaviors related to salt.

1. Do you know how much blood pressure in adults can be diagnosed as hypertension?
   (1) 140/90mmHg
   (2) 130/80 mmHg
   (3) 120/80 mmHg
   (9) I don’t know
2. Do you know what diseases hypertension can cause (multiple choices)?
   (1) Stroke
   (2) Coronary heart disease
   (3) Kidney disease
   (4) Hypertensive heart disease
   (5) Eye diseases
   (9) I don’t know
3. Do you know which factors are related to the prevalence of hypertension (multiple choices)?
   (1) Overweight or obese
   (2) Long-term excessive drinking
   (3) Long-term high-salt diet
   (4) Have a family genetic history
   (5) Hyperglycemia and hyperlipidemia
   (6) Age
   (7) Stress
   (9) I don’t know
4. Do you know that adults should not eat more than a few grams of salt per person per day?
   (1) 2g
   (2) 6g
   (3) 9g
   (4) 12g
   (9) I don’t know
5. Do you know that eating less salt helps lower blood pressure?
   (1) Know
   (2) I don’t know

6. Do you know what diseases may result from eating too much salt (multiple choices)?
(1) Hypertension
(2) Stroke
(3) Myocardial infarction
(4) Kidney disease
(5) Gastric cancer
(6) Osteoporosis
(9) I don’t know
7. Do you think you eat too much salt?
(1) Not much
(2) Moderate
(3) Too much
8. Do you plan to eat less salt after knowing the dangers of eating too much salt?
(1) Intend to
(2) Not intend to
9. Has your family ever used a salt-restriction spoon?
(1) Used
(2) Not used
10. Do you know how to use salt-restriction spoon correctly?
(1) Know
(2) I don’t know
11. Do you usually use the SRS correctly in daily cooking?
(1) Yes
(2) No
12. Do you think low-salt diet should be promoted among the crowd?
(1) Should
(2) Should not
13. Have you ever been promoted or educated on a low-salt diet?
(1) Accepted
(2) Not accepted
14. Have you ever promoted the knowledge of low-salt diet to the people around you?
(1) Promoted

(2) Not promoted

15. Do you think a low-salt diet affects the taste of food?

(1) Great influence

(2) Has a certain influence, but can accept

(3) No effect

16. What kind of people do you think should pay special attention to low-salt diet (multiple choices)?

(1) Hypertensive patients

(2) Patients with stroke

(3) Patients with coronary heart disease

(9) I don't know

17. Have you paid attention to the salt / sodium content of the food when purchasing processed food?

(1) Followed

(2) Not followed

18. Do you think processed foods should be labeled with the salt / sodium content of the product?

(1) Should

(2) Should not

19. Do you think that labeling the salt / sodium content of processed foods will help you choose low-salt foods?

(1) Yes

(2) No

20. What is your attitude towards a low-salt diet?

(1) For

(2) Against

21. Have you taken active salt reduction measures yourself?

(1) Yes

(2) No

22. Have you heard of low-sodium salt (alternative salt)?

(1) Yes

(2) No

23. Do you know that low-sodium salt helps control blood pressure compared to regular table salt?

(1) Know

(2) I don’t know

24. Have you used low-sodium salt?

(1) Used

(2) Not used
